# Estimation of Cold Stress, Plant Age, and Number of Leaves in Watermelon Plants Using Image Analysis

**DOI:** 10.3389/fpls.2022.847225

**Published:** 2022-02-18

**Authors:** Shona Nabwire, Collins Wakholi, Mohammad Akbar Faqeerzada, Muhammad Akbar Andi Arief, Moon S. Kim, Insuck Baek, Byoung-Kwan Cho

**Affiliations:** ^1^Department of Biosystems Machinery Engineering, Chungnam National University, Daejeon, South Korea; ^2^Department of Smart Agriculture Systems, Chungnam National University, Daejeon, South Korea; ^3^Environmental Microbial and Food Safety Laboratory, Agricultural Research Service, United States Department of Agriculture, Beltsville, MD, United States

**Keywords:** chilling stress, phenomics, image processing, morphological traits, leaf count, plant age

## Abstract

Watermelon (*Citrullus lanatus*) is a widely consumed, nutritious fruit, rich in water and sugars. In most crops, abiotic stresses caused by changes in temperature, moisture, etc., are a significant challenge during production. Due to the temperature sensitivity of watermelon plants, temperatures must be closely monitored and controlled when the crop is cultivated in controlled environments. Studies have found direct responses to these stresses include reductions in leaf size, number of leaves, and plant size. Stress diagnosis based on plant morphological features (e.g., shape, color, and texture) is important for phenomics studies. The purpose of this study is to classify watermelon plants exposed to low-temperature stress conditions from the normal ones using features extracted using image analysis. In addition, an attempt was made to develop a model for estimating the number of leaves and plant age (in weeks) using the extracted features. A model was developed that can classify normal and low-temperature stress watermelon plants with 100% accuracy. The R^2^, RMSE, and mean absolute difference (MAD) of the predictive model for the number of leaves were 0.94, 0.87, and 0.88, respectively, and the R^2^ and RMSE of the model for estimating the plant age were 0.92 and 0.29 weeks, respectively. The models developed in this study can be utilized in high-throughput phenotyping systems for growth monitoring and analysis of phenotypic traits during watermelon cultivation.

## Introduction

Watermelon (*Citrullus lanatus*) is a highly nutritious fruit comprised of 93% water with small quantities of protein, fat, minerals, and vitamins. It is widely considered a functional food, thus contributing to its widespread consumption around the world (Assefa et al., 2020). Watermelon is a member of the cucurbit family (*Curcurbitaceae*), which are chill-sensitive plants that are native to subtropical and tropical regions around the world. There are four main cucurbit crops, namely cucumber, watermelon, melon, and squash. Of these main crops, watermelon has the highest worldwide consumption (Wehner et al., 2020).

Watermelon plants are characterized by big leaves, long, and thin hairy stems that can grow up to 10 m long with branched coiled tendrils at the nodes and yellow flowers. Its leaves are green, with blades of about 20 × 20 cm, pinnately lobed, and usually divided into three to five pairs of lobes. Its growth habit is a long trailing vine, due to which the plants are usually grown at a wide spacing (Aruna et al., 2016). Watermelons are mainly grown in tropical and subtropical climates and require a warm growing season of 75–95 days from planting to harvesting. While the optimum growth temperature for watermelons ranges from 21 to 29°C, they can tolerate a minimum of 18°C and a maximum of 32°C (Noh et al., 2013; Shirani Bidabadi and Mehralian, 2020). Watermelons are highly temperature sensitive depending on the growth stage. In the early stages of plant growth, 25°C is optimal and growth has been observed to stop at 10°C. Below temperatures of 13°C, flowering does not occur and above 45°C only mature plants can survive (Noh et al., 2013).

Plants are vulnerable to a wide range of physical and chemical variables, including low and high temperatures, insufficient or excessive water, high salinity, heavy metals, and ultraviolet (UV) exposure, among others. These stresses, known collectively as abiotic stresses, pose a danger to agriculture and the ecosystem, accounting for significant crop production loss. In watermelon plants, abiotic stresses caused by temperature extremes (Rivero et al., 2001; Shirani Bidabadi and Mehralian, 2020), water stress (Yoosefzadeh Najafabadi et al., 2018), salinity stress (Yetişir and Uygur, 2009; Li et al., 2017), etc., are the most prevalent. In the watermelon plant life cycle, both reproductive and vegetative stages are negatively affected by low temperature stress (Nishiyama, 1970; Shirani Bidabadi and Mehralian, 2020). During reproductive development, low temperature stress can delay flowering and induce flower abscission, pollen sterility, pollen tube shortening and distortion, and reduced fruit set, which lowers yield (Waraich et al., 2012). The effects of cold stress on the reproductive stage have important economic and social effects since the products of this stage are the source of food supply (Thakur et al., 2010; Zinn et al., 2010). At the vegetative development stage, low temperatures can cause a reduction in stomatal conductance and leaf water content, therefore resulting in smaller leaves and shoots (Rodríguez et al., 2015). Collectively, these stress responses reduce fruit yield and quality, which has negative economic and nutritional impacts (Lu et al., 2003; Taylor et al., 2003).

In climates with short warm seasons, seeds are sown in growth chambers and transplanted into the field or protective structures after 3–4 weeks (Wehner et al., 2020). The largest protective structures for commercial watermelon production in non-tropical climates are glasshouses (greenhouses). These have systems that control lighting, shading, heating and cooling, ventilation, humidity, and carbon dioxide concentration. Due to the temperature sensitivity of the watermelon plants, temperatures must be closely monitored in the controlled environments. It is necessary to understand plant responses to temperature stresses to improve management within the controlled environments. Studies have found that immediate plant morphological responses to these stresses include reductions in leaf size, number of leaves, and plant size (Bismillah Khan et al., 2015; Fahad et al., 2017).

Plant morphological studies involve a detailed study of vegetative and reproductive plant structures that can be used to make comparisons between species, identify different varieties, or study plant responses to stimuli (Wyatt, 2016). Some of the key morphological features relevant to plant morphological studies are leaf shape, size, color, texture, angle, and volume. In the shoot system, leaves experience significant changes in morphology in response to the environment that can be easily observed (Yang et al., 2015). Leaf morphological features can be important determinants of plant performance because leaf size and shape influence key plant productivity processes such as photosynthesis, stomatal conductance, and transpiration efficiency (An et al., 2017). In studies involving morphological feature analysis of plants, some key features measured include plant leaf length, width, angle, diameter, perimeter area, and volume (Harish et al., 2013). Leaf morphological features are useful for plant recognition, identification, classification, and disease identification and classification (Aptoula and Yanikoglu, 2013; Ramcharan et al., 2017, 2019; Kumar et al., 2019; Tan et al., 2020).

Image analysis has found wide application in various domains of science. The image analysis workflow consists of image capture, preprocessing, feature extraction, and analysis. In plant studies, imaging techniques and analysis have the advantage of being non-destructive and able to extract intricate information that can be used to analyze biological patterns of plant growth (Nabwire et al., 2021). The application of image analysis in morphological studies has been done to automate plant recognition tasks (Aptoula and Yanikoglu, 2013; Kumar et al., 2019), classification of plant leaves using leaf shape feature extraction techniques (Manik et al., 2016), automation of plant classification systems (Harish et al., 2013), and development of leaf disease detection and diagnosis systems (Jagtap and Hambarde, 2014). Specifically, image analysis has been applied in cold stress response classification in maize plants (Enders et al., 2019), drought and heat stress tolerance screening in wheat (Schmidt et al., 2020), weed growth stage estimation (Teimouri et al., 2018), and leaf counting in *Arabidopsis* using deep learning (Aich and Stavness, 2017). These studies achieved acceptable results, however there have been no studies that have applied image analysis to identify cold stress plants, estimate leaf count, and plant age in watermelon plants.

High throughput plant phenotyping (HTPP) systems are useful for quantifying/estimating the effects of exposure to sub optimal conditions (temperature, water, etc.) on individual plant through estimating various plant characteristics. The data from such systems is useful for making comparisons between plant species, identifying varieties, or study plant responses to stimuli. This information is useful for research and in decision making. Therefore, the objective of this study was to extract and analyze the morphological features (relating to form, structure, texture, and color) of watermelon plants using image analysis. The features were used to develop a model for prediction of cold stress condition of the plants and determination of the number of leaves and plant age. The models developed in this study can be utilized in high-throughput phenotyping systems for growth monitoring and analysis of phenotypic characteristics (such as number of leaves, plant age) during watermelon cultivation.

## Materials and Methods

### Dataset

The watermelon seed samples were acquired from Partner seed (Gimje, Jeollabuk-do, South Korea). Four test varieties, namely, DAPCT, PI482261, DAP, and 45NC, as defined by the seed company, were used for this study. A total of 128 seeds were used, including 12 from the PI482261, 16 from the 45NC, 52 from the DAP, and 48 from the DAPCT variety. The seeds were planted in a container in individual cells and placed in a growing chamber maintained at 28°C and 70% relative humidity to ensure germination. After 2 weeks, the seedlings were transplanted into individual cylindrical pots (12 cm diameter, 11 cm height) with porous bases and transferred to the growing chamber that was reserved for this experiment. A nutrient mixture called “Mulfuresiriz” from Daeyu company limited (Seoul, Gyeonggi-do, South Korea) used in hydroponics was applied equally (concentration of 4 ml of nutrient mixture per liter of water) to all the pots at the start of the experiment.

### Experiment Design

The experiment design for this study was based on the growth stages of watermelon plants. Data collection was done weekly between the 2nd and 5th week (seedling to flowering stage of watermelon plants) at total of 4 weeks (week 2, 3, 4 and 5). This is because at less than 13°C flowering will not occur; however, after flowering and fruit set, temperatures greater than 14°C have no significant effect on plant growth (Noh et al., 2013). The watermelon plants were separated in two groups, the normal group (plants grown in optimum growth conditions), and the stressed group (plants grown in cold stress conditions). A total of 10% of the plants from each variety were stressed each week. The plants to be considered for the stress group were selected using random numbers to eliminate bias. The growth temperature considered for the control (normal) and the stressed group in this study were as detailed in Table 1. The plants in each group were grown in separate chambers that were both maintained at a relative humidity of 70% for the entire growth period. The lighting used for both growth chambers was 15,000 lux intensity, 6,500K color temperature for the simulated day hours and no lights for the simulated night hours. The concentration of carbon dioxide gas was maintained at 700 ppm for both growth chambers. Both the normal and stressed group plants followed the same watering regime, which was done after every 2 days. During watering, the plant pots were soaked in 2 cm deep pure water for 4 h to allow time for water to percolate into the soil.

**TABLE 1:** Plant growth conditions and weekly stress plan.

Plant condition	Number of plants				Optimal temperature	Growth temperature	
			
	Week 2	Week 3	Week 4	Week 5		Day (16 h)	Night (8 h)
Control group	128	116	104	92	20–30°C	28°C	21°C
Stress group	0	12	24	36	15°C	15°C	10°C

### Data Collection

**FIGURE 1:**
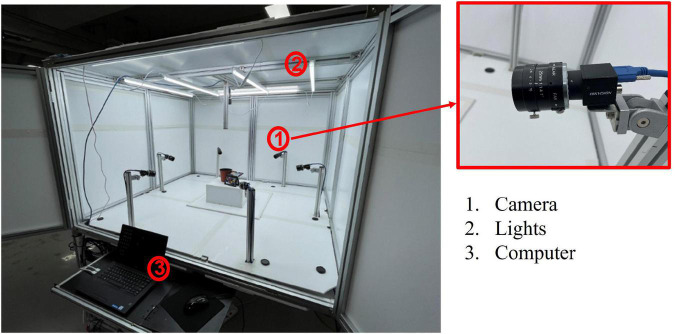
System for data collection.

Image data collection was done using a multi-camera system (Figure 1). The system specifications are detailed in Table 2. The reason for the multiple camera setup was to capture more views of the plant from which to choose when extracting consistent and representative morphological features. The target field of view (FOV) for the cameras was 32 × 27 cm (enough to cover the entire watermelon plant), therefore the camera-to-sample distance for the system was 60 cm to accommodate the FOV. The cameras were set up with the same angle, distance, aperture, and exposure time (10,000 μs). Color calibration was done during data collection to compensate for variations in color channel values, aperture opening, and manufacturing tolerances that can result in varying camera color signatures. Color calibration was done by taking images of the standard X-Rite color chart, extracting the color values of the patches, and finding the best transform matrix that maps the resultant color values with their respective reference values. The resultant color correction matrix was then used to transform the images taken from the cameras to their true color (Sunoj et al., 2018).

**TABLE 2:** System specifications.

System dimensions	Camera specifications	Lights
Width: 160 cm Length: 168 cm Height: 107 cm	Name: HIKVISION MV-CA050-20UC Type: 5MP 1” CMOS USB3.0 Resolution: 2592 × 2048 Lens: 25 mm lens Variable aperture: f/1.4 to f/16 (fixed to f/8) Number: 7 cameras	Type: D65 White LED Power: 15W Quantity: 6 lights

One camera was set up at the top of the system to capture the top view image of the plant, while the other six cameras were set up to capture the side view of the plant at 60 degree intervals from each other.

Reference data, which include number of leaves, plant age (weeks), and stress condition (control or stressed), were recorded for each plant every week after image data collection.

### Thresholding/Background Removal

**FIGURE 2:**
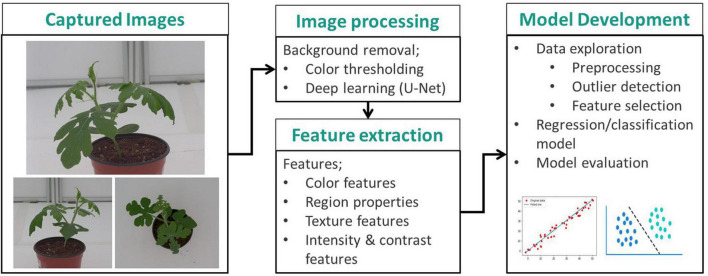
Data analysis workflow.

For the analysis, three images were selected from the top view camera and cameras at 0 and 60 degrees. The image views were labeled image 1, image 2, and image 3, corresponding to the 0-degree, 60-degree, and top view image, respectively. A summary of the data analysis workflow for this study is shown in Figure 2.

**FIGURE 3:**
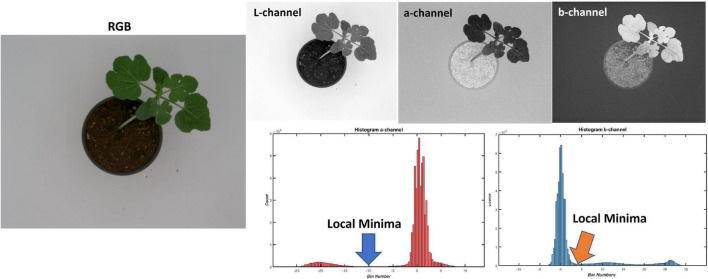
Summary of conventional image processing background removal algorithm.

Background removal was carried out using two methods to define the region of interest (the plant) for further processing. Initially, it was carried out using conventional image processing. This involved conversion of the image from RGB to the CIELAB color space. An analysis of the histograms of each channel resulted in a necessity to keep all pixels below the local minima in the “*a”* channel and above the local minima in the “*b”* channel (Figure 3). Since these local minima (for both channels) varied for each image, a search algorithm that automatically determines the position of the local minima in a predefined range was used. The determined position values were used as thresholds to make two binary images, which were then combined to create the watermelon plant mask.

Deep learning was also applied for background removal using U-Net, a network commonly used for image segmentation. It can work with few images and give accurate segmentation results. The network does not have fully connected layers and uses the pixels in the segmentation map whose full context is available in the input image. It uses successive layers with pooling layers replaced by upsampling layers therefore increasing the output resolution. The upsampling section of the network has many feature channels that enable the network to propagate context information into the higher resolution layers. This makes the expansive path symmetric with the contracting path and gives the architecture its characteristic U-shape (Ronneberger et al., 2015). Although U-Net was originally developed for application to biomedical images, it has been applied in various domains of science, including audio signal source separation and satellite imagery (Stephan and Santra, 2019).

The network was trained on 830 images with a ratio of 7:2:1 for the training, validation, and test datasets, respectively. The input image size for the network was 512× 512× 3 and the output image size was 512×512. The network was trained for 100 epochs using Adam optimizer, mini-batch size of 8, learning rate of 0.001, drop factor of 0.9, and drop period of 5.

### Feature Extraction

#### Color Feature Extraction

The color features considered for this study were extracted from four color spaces (RGB, HSV, CIELAB, and YCbCr). The color space suitable for an application is selected based on the acquisition setup. While HSV and CIELAB represent colors in a format closer to human vision, CIELAB has the advantage of being able to detect small differences in color and is device independent. YCbCr is suitable for image/video data compression. The color components are represented by coefficients of the three colors depending on the selected color space. They are extracted by conversion of the image to the desired color space and averaging the color values in each component (Kavitha and Suruliandi, 2016). Color feature extraction was done by conversion of the images from RGB to the major color spaces: HSV, LAB, and YCbCr. The average value of the color channels in each of the color spaces was computed to extract the color features – in total 12 feature values for each image.

#### Shape-Based Feature Extraction

Shape-based feature extraction is carried out to extract features that describe the shape and size of a region of interest in an image. Shape and size parameters, sometimes referred to as region properties, quantify the shape of the region depending on the requirements of the image processing task (Mingqiang et al., 2008). The region properties of the region of interest (Table 3) were extracted from the resultant mask from the image segmentation process. These amounted to a total of 30 feature values for each image.

**TABLE 3:** Region properties used for shape-based feature extraction.

Parameter	Description
Area	The number of pixels in the selected region of the image.
Bounding box	The rectangle that contains every point in the selected region.
Major axis length	The length of the line connecting the base point to the tip of the leaf.
Minor axis length	The length of the line perpendicular to the major axis.
Centroid	The center of mass of the region being analyzed.
Solidity	The ratio of the leaf area to the area of the convex hull. This is useful for measuring the density of the region.
Perimeter	The length of the external shape of the region being analyzed.
Circularity	A measure that describes the roundness of an object.
Convex hull	This is the smallest convex polygon that contains the selected region.
Equivalent diameter	A measure of the diameter of a circle that has the same area as the region of interest.
Eccentricity	The ratio of the distance between the foci of an ellipse that has the same second-moment as the region of interest and the length of its major axis.
Maximum Feret diameter	The maximum distance between two boundary points on the antipodal vertices of the convex hull.
Minimum Feret diameter	The smallest distance between two boundary points on the antipodal vertices of the convex hull.
Extent	The ratio of pixels in the region of interest to the pixels in the bounding box.

#### Texture Feature Extraction

Texture can be defined as the surface quality of a region of interest. In image processing, texture is analyzed based on the variations in the gray tone values extracted from an image (Metre and Ghorpade, 2013). Texture features are commonly extracted using Gray Level Co-occurrence Matrix (GLCM) to find symmetry in the texture in an image (Hu and Ensor, 2019). It is based on the occurrence of the gray level configuration and measures the spatial relationships between pixels to infer texture information (Ehsanirad and Sharath, 2010). Haralick texture features are derived from the GLCM (Haralick et al., 1973). They consist of 14 statistical features, which include autocorrelation, contrast, cluster prominence, cluster shade, correlation, etc. For this analysis, four properties were extracted using the GLCM, namely contrast, correlation, energy, and homogeneity. Using a Haralick distance of 3, 28 texture feature values were extracted for each image.

Additionally, other texture feature extraction methods, including local binary patterns (LBP), discrete cosine transform (DCT), Fourier descriptors, and Gabor features, were used. These features were extracted using the BALU toolbox in MATLAB (Mery, 2011).

Local binary patterns (LBP) are generated by the best matching pattern in the image and are responsive to edges, lines, spots, and flat areas, whose distribution is estimated by the occurrence histogram. They are key texture properties and provide most patterns in observed textures. LBP features derive their name from the functionality of the LBP operator *LBP*_*P*,*R*_, whereby the threshold of the local neighborhood is determined at the gray value of the center pixel in a binary pattern (Ojala et al., 2002). LBP features address the challenge of non-uniformity of textures due to variations in orientation, scale, or resolution of an image. For each image, a uniform LBP operator was applied with eight neighborhood pixels and one vertical and horizontal division. This resulted in 59 LBP feature values for each image.

Discrete Cosine Transform (DCT) is a unitary image transform that transforms the image from the spatial domain to the frequency domain. Unitary transformations are useful in image processing in that they preserve the length of the vector and pack a large fraction of the mean energy of an image into a few transform coefficients, allowing for the preservation of feature information (Jain, 1989; Kumar and Bhatia, 2014). DCT separates the image into parts of varying importance depending on the image visual quality. It gives coefficients that are both local and global features. DCT is a popular feature extraction transform in terms of its compact feature representation and computational complexity arising from its data independent nature (Chadha et al., 2011). For this study, a vertical and horizontal resize of 64 and frequency of 2 were applied to each image to extract four DCT coefficients.

Fourier-based feature extraction involves the transformation of the image from the spatial to the frequency domain and has the advantage of eliminating noise that occurs at higher frequencies. Using this technique, a spectrum of texture is obtained using a Fourier transform. Local and global texture feature descriptors are obtained from the spectrum. Fourier spectrum descriptors describe the direction and formation of texture patterns (Hu and Ensor, 2019). For this study, a frequency of 2 was applied to each image to generate 8 spectral peaks and 16 texture descriptors.

Gabor feature extraction method extracts the Gabor features of an image using a Gabor filter function. The Gabor filter function is useful in texture analysis where texture is non-uniform (Kumar and Pang, 2002). They extract local pieces of information that are combined to recognize the object of interest, making this method one of the superior methods for complex tasks such as facial recognition (Kamarainen, 2012). Eight rotations and dilations were applied to each image with a frequency ranging from 0.1 to 2 to generate 19 Gabor feature values.

#### Intensity-Based Feature Extraction

Other features extracted include intensity and contrast features. Intensity-based feature extraction extracts the color intensity values for each pixel (Sabrol and Kumar, 2016). Contrast measures the differences in brightness levels between the light and dark areas of an image (Chen et al., 2019). The parameters extracted in this feature extraction method include, but are not limited to, maximum intensity – the intensity value of the pixel with the greatest intensity in the region of interest, mean intensity – the average intensity of all the intensity values in the region of interest, minimum intensity – the intensity value of the pixel with the lowest intensity in the region of interest, and the weighted centroid – the center of the region of interest based on intensity values. Intensity features were extracted from the green channel – a total of six feature values from each image. Contrast features were similarly extracted from the green channel image resulting in five feature values. Both functions were inherited from the MATLAB BALU toolbox (Mery, 2011).

The resultant features (Table 4) were concatenated horizontally for each watermelon plant sample. The resulting features from each plant were concatenated vertically, resulting in a data matrix (rows = sample, columns = features), which was used for modeling the phenotypic traits.

**TABLE 4:** Total number of features extracted for each watermelon plant.

Feature type	Number of features	Remarks
Region properties	30	Extracted from mask image
Color features	12	Average color values
Texture features	138	Sum of all the texture features
Other features	11	Including contrast and intensity features
Total for each image	191	Number of features extracted per image
Total for three images	573	Total number of features extracted per plant

### Data Analysis and Model Development

#### Feature Preprocessing

Feature extraction methods use different formulae and conventions and therefore output feature values are of varying magnitudes. Preprocessing of the features is necessary before data analysis to enhance the features, remove noise that may result from intensity variations in the image, and standardize the ranges of feature values. For this reason, a normalization vector was applied using min–max normalization (Patro and Sahu, 2015), which resulted in values ranging between 1 and 0.

#### Outlier Detection

Outliers are extreme data points that deviate from other observations of the data and may indicate experimental errors, data processing errors, or variability in measurements (Wang et al., 2019). The outliers in the extracted dataset were likely caused by misdetections during feature extraction. Outliers are bound to exist and can influence the model development process and overall model performance.

To remove outliers from the data, two steps were followed:

(1) Computation of principal component analysis (PCA) of the data, followed by extraction scores of the first five PC (representing more than 90% of variance in the data);

(2) Use of robust multivariate dispersion algorithm (Olive and Hawkins, 2010) on the extracted scores to determine which samples are outliers and which are inliers.

#### Feature Cleaning and Selection

Feature selection is usually done to select a group of features from the original set that contain accurate distinguishing information of one object from another for accurate predictions in the model (Kumar and Bhatia, 2014). It may consist of feature cleaning, where features that are redundant and those that contain little or no information are removed from the feature set. The decision to remove some features is subjective depending on the parameters being predicted by the model. The removal of some features that contain noise may compromise prediction accuracy in cases where they contain valuable information for the prediction of some parameters (Tallón-Ballesteros and Riquelme, 2015).

Due to the many features extracted from the image data, it is likely that the data contained irrelevant features possibly due to noise or redundancy/collinearity in the data. To reduce the effects of these irrelevant features, feature cleaning and a feature selection algorithm called sequential feature selection (SFS) were used to find relevant features. The feature cleaning algorithm was used to eliminate constant and correlated features. The SFS algorithm searches for the linear combinations of features that best correlate with the responses (Pudil et al., 1994). Both the feature cleaning and SFS algorithms used are available in the MATLAB BALU toolbox (Mery, 2011). The resultant few features were used to develop the final models.

#### Model Development

After the feature cleaning and selection process, a few important features were retained. Depending on the phenotypic trait, a classification or regression model was developed. In the model development process, 70% of the data were used for model calibration (implemented with fivefold, k-fold cross validation) and 30% were used to test the resultant models.

Linear discriminant analysis (LDA), a commonly used technique for classification and dimensionality reduction (Varmuza and Filzmoser, 2009), was used to develop a model for discriminating between normal (control) and cold-stressed watermelon plants. Plants that belong to the control group were labeled 1 while all stressed plants (1, 2, and 3-week stressed plants) were labeled 2.

Multiple linear regression (MLR) is known for its simplicity in finding correlations between multiple variables and responses (in this case number of leaves and plant age). The MLR algorithm is known to fail with high-dimensional data and highly correlated data (Marill, 2004). However, due to the reduced number of variables after SFS variable selection (reduction to less than 40 features), applying MLR was sufficient for this study to develop models for prediction of number of leaves and plant age.

#### Model Evaluation

The performance of the LDA model(s) developed for classifying normal from cold-stressed plants was evaluated using classification accuracy and confusion matrix (which shows the specificity and precision of the model) for both the calibration and prediction sets (Visa et al., 2011; Raschka, 2018).

The performance of the MLR models developed for predicting number of leaves and plant age was tested using the goodness of fit criteria, including root-mean-square error (RMSE) and coefficient of determination (R^2^) for the calibration and prediction sets (Zhou and Bovik, 2009; Chai and Draxler, 2014). The best models should have R^2^ values close to 1, and RMSE values close to zero.

Finally, tests were carried out on the results to evaluate and find optimum conditions. These tests include:

(1) Analysis of the composition of the selected features to determine the most relevant and abundant features and image view from whence most features are extracted;

(2) Testing the results using 1, 2, and 3 image views to determine the most suitable number of images to be used for predicting the phenotypic traits.

## Results

### Image Data

**FIGURE 4:**
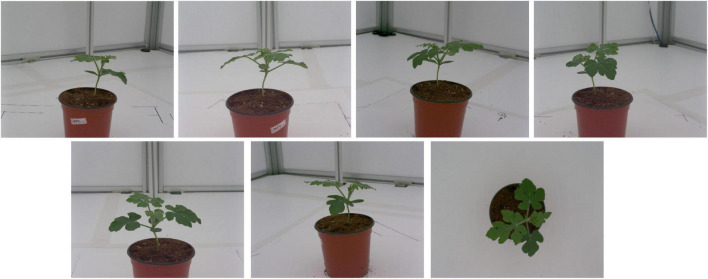
Watermelon plant images captured using the data collection setup.

Using the data collection system setup, the seven cameras each captured one image for each watermelon plant sample placed at the center of the system. The captured images (Figure 4) were then saved in a specified directory in a portable network graphics (PNG) format. The images were later fed into an image analysis pipeline to estimate the phenotypic traits of the watermelon plants.

### Background Detection

**FIGURE 5:**
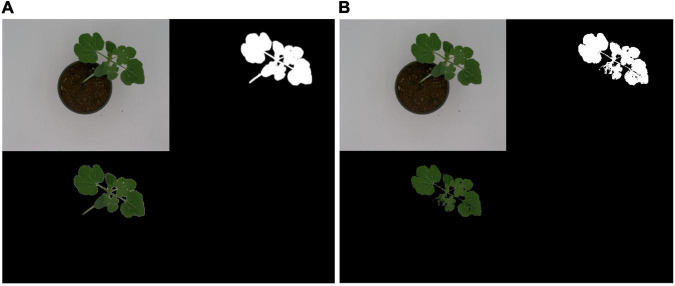
Comparison of watermelon plant background segmentation using **(A)** U-Net and **(B)** a conventional image processing algorithm.

The results of the two methods used for background removal showed that U-Net performed better than the conventional image processing algorithm (Figure 5). The less-than-pristine performance of the conventional image processing-based algorithm was due to of the poorly handled variances that existed in the data caused by inter-image intensity differences due to sample color/intensity variances. From these results, U-Net background removal was used for segmenting the watermelon plant from the background scene.

### Discrimination of Stressed and Non-stressed Plants

**FIGURE 6:**
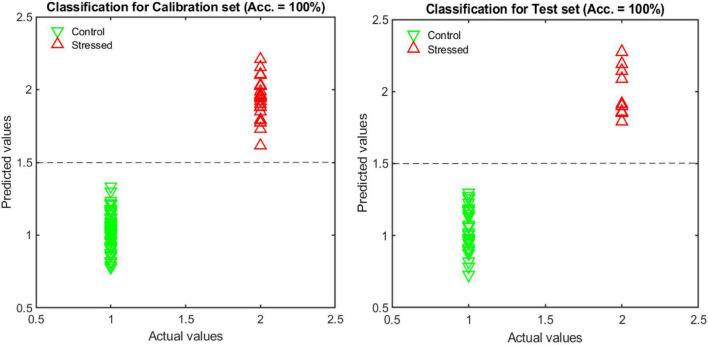
Classification results for normal and stressed plants using three images.

Results of the LDA classifier for classification between normal and cold-stressed plants resulted in 100% classification accuracy both on the calibration and test data set (Figure 6).

**FIGURE 7:**
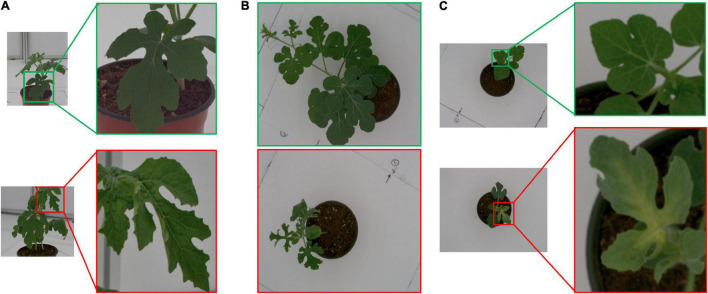
**(A)** Textural difference between a normal (top) and stressed (bottom) plant at week 4. **(B)** Size difference between a normal (top) and stressed (bottom) plant at week 5. **(C)** Color difference between a normal (top) and stressed (bottom) plant at week 3.

The reason for the clear discrimination is because of the clear differences (in size, texture, and color) between the normal and stressed plants (Figure 7). An analysis of the features selected for classifying normal and cold-stressed plants revealed that 68.2, 18.2, 4.5, and 9.1% belonged to texture, region properties, color, and other features, respectively (Table 5). Texture features (describe plant texture) and region properties (describe plant shape and size) constituted more than 86% of the selected features and thus contributed more to the classification between normal and stressed watermelon plants.

**TABLE 5:** Selected features for classification of plant stress condition.

Features		Number of features	Percentage
Texture	LBP features	5	68.2%
	Haralick features	2	
	Fourier features	3	
	DCT coefficients	2	
	Gabor features	2	
Region properties	Feret properties	2	18.2%
	Euler Number	1	
	Orientation	1	
Color		1	4.5%
Others	Contrast	2	9.1%
Total		21	

The predominant texture features in the analysis were the DCT, LBP, Haralick, Gabor, and Fourier descriptor features. Further analysis established that features from image 2 (60-degree view image, 50% of the selected features) were more abundant, followed by those from image 3 (top view image, 27.3% of the selected features) and image 1 (0-degree view image, 22.7% of the selected features).

The classification model development was repeated using features extracted from two images and one image, and a comparison was made to find out which number of image views is most suitable. The results show a slight reduction in model precision and accuracy as the number of images is reduced (Table 6), though not significantly different when using two or three images. However, the smaller the number of images used, the more complex the resultant classification, requiring more features to make a reliable classification. These results show that a minimum of two images are required for 100% classification accuracy.

**TABLE 6:** Comparison of classification results for normal and stressed watermelon plants using features from three-view, two-view, and one-view images.

Number of Images	Calibration					Test					Selected features	Outliers
		
	TP	FP	TN	FN	Acc. (%)	TP	FP	TN	FN	Acc. (%)		
3 images	62	0	23	0	100	27	0	9	0	100	21	1
2 images	61	0	23	0	100	27	0	9	0	100	22	2
1 image	61	0	22	0	100	24	1	9	0	98	26	4

### Leaf Counting

**FIGURE 8:**
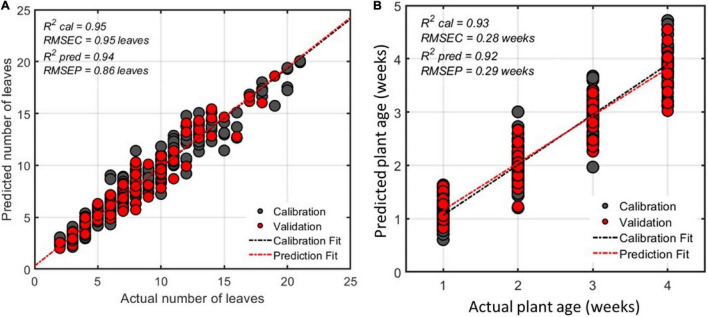
Regression plot from model for estimating number of leaves of watermelon plant **(A,B)** watermelon plant age for all varieties estimated using 21 selected features.

Of the 573 features from the feature extraction process, 21 features were selected using the SFS algorithm and were used to estimate the leaf count of the watermelon plants. The number of leaves detected using the morphological features was correlated with the real number of leaves in the corresponding plants. The R^2^, RMSE, and mean absolute difference (MAD) values achieved during prediction were 0.94, 0.97 leaves, and 0.88 leaves, respectively (Figure 8).

### Plant Age Estimation

For plant age estimation, number of weeks was used as the reference information since image data was collected every week for 4 weeks. Using the extracted features, 15 features were selected using the SFS feature selection algorithm and a regression model using MLR was developed to find a correlation between the selected features and the normal/control plant age in weeks. The performance of the model for predicting watermelon plant age was found to have R^2^ and RMSE values of 0.92 and 0.29 weeks, respectively (Figure 8). This model was developed for all four watermelon varieties. However, using data from the individual watermelon varieties resulted in higher model performance in prediction (Table 7).

**TABLE 7:** Multiple linear regression model performance based on all and individual watermelon varieties.

Varieties	Calibration		Test		Selected features	Outliers
		
	R^2^	RMSE	R^2^	RMSE		
All varieties	0.93	0.28	0.92	0.29	15	5
DAP	0.98	0.15	0.98	0.15	18	2
DAPCT	0.98	0.12	0.97	0.17	18	5
PI482261	1.00	0.02	0.99	0.04	14	1
45NC	1.00	0.03	1.00	0.05	18	1

## Discussion

### Discrimination Between Stressed and Non-stressed Plants

Temperature stress is a significant challenge to agricultural production. Extreme changes in temperature that deviate from a plant’s optimal growth temperature range restrict plant metabolism, growth, and development (Yadav, 2010; Ding et al., 2019). In non-tropical climates, watermelon plants are cultivated in controlled environments where the plants are closely monitored to prevent the effects adverse temperature changes. Plants that are exposed to cold stress show symptoms including retarded growth, yellowing of leaves (chlorosis), wilting and reduced leaf expansion after 48 h of exposure (Yadav, 2010). Previous studies that used image analysis to assess cold stress in plants focused on variety performance comparison (Enders et al., 2019), and chilling stress injury classes (Dong et al., 2019). However, none of these studies attempt to classify cold stressed plants from normal ones.

This study results demonstrated the possibility of classification of cold-stressed watermelon plants from normal plants and determination of some phenotypic traits based on image analysis. The classification model was able to distinguish the stressed plants after 1 week of exposure to stress conditions from the normal plants. Analysis of the selected features showed that texture was most important for the classification (Table 5). This is because during cold stress, the leaf shape of the plants is shriveled at the edges, causing a change from a smooth hairy texture to a coarser rough texture (Figure 7).

Similarly, the stressed plants were smaller in size than normal plants because plant growth is stunted during the cold stress spell (Figure 7). This was clearly seen in the plant image data resulting in a high feature importance for the shape-based features (region properties). This is because cold stress disrupts bio-energetic processes, causes changes in metabolism, and contributes to damage to cellular structures, hence the stunted growth and shriveled leaves (Korkmaz and Dufault, 2001; Staniak et al., 2021).

These clear differences in the image data resulted in a distinct classification between cold-stressed watermelon plants from normal ones regardless of the age and variety. A further analysis into the number of image views required for extraction of morphological features resulted in a minimum requirement of two image views (Table 6). Using one image-view to extract morphological features required a larger feature set of 26 features and achieved a lower classification accuracy. This is because of occlusion of plant leaves using one image view that was alleviated by using multiple views.

### Leaf Counting

Over the duration of the data collection period, it was noted that the watermelon plants exposed to stress were stunted and had a lower leaf count compared to the normal plants. This signifies the importance of counting the number of leaves as a phenotypic trait in plant monitoring to determine plant health alongside other traits. The leaf counting task is specialized and requires a new model to be developed for each plant due to variations among species. Similar to this study, Pape and Klukas (2015) used a few geometric features to carry out the leaf counting task for *Arabidopsis thaliana* and tobacco plants. This study achieved comparable results to theirs using geometric as well as texture features to carry out leaf counting for watermelon plants which, to the best of our knowledge, has not been done in previous studies.

Similar results to ours have been obtained in previous studies using DL algorithms for the leaf counting task (Aich and Stavness, 2017; Ubbens and Stavness, 2017). However, the use of deep learning requires the annotation of each leaf in a plant image to generate a training dataset from which the algorithm learns and can then make accurate predictions of leaf counts from new images. This requires precise delineations, the acquisition of which is time-consuming and sensitive to the arrangement of leaves (Giuffrida et al., 2018). The model in this study attempted to overcome this challenge with the extraction and application of morphological features from images, which resulted in superior results for leaf counting that are comparable to previous studies that have used DL algorithms.

### Plant Age Estimation

The sensitivity of watermelon plants to temperature stress varies depending on the age of the plant. The adverse effects of temperatures below 13°C can be seen before the flowering stage and plants that are subject to temperatures greater than 14°C beyond the flowering stage do not experience significant cold stress effects. This was the basis for the experimental design of this study. Estimation of the plant age can be done based on the number of leaves and tillers (Girma et al., 2007; Teimouri et al., 2018). For this study, the plant age was determined based on the number of weeks from the 2nd week (seedling stage) to the 5th week (flowering stage) i.e., for week 2, 3, 4, and 5.

The regression results (Table 7) showed distinct predictions of the age of the plants from all four varieties. A further analysis of plant age prediction for individual varieties resulted in better prediction performance. Because of the differences between the watermelon varieties, data from a single variety is more homogenous and subject to less variation compared to data from all the watermelon varieties, resulting in better model performance. This is consistent with the phenomenon of intraspecific variation that accounts for the phenotypic and genotypic variation within a species (des Roches et al., 2018). This phenomenon influences models that are used for estimating the watermelon plant age.

In summary, a simple plant-to-sensor system was developed that can identify cold stressed watermelon plants and additionally estimate plant characteristics including leaf count and plant age. This study applies an image analysis pipeline (image processing, feature detection, extraction, and selection) on the captured watermelon plant images to identify cold stressed plants and estimate leaf count and plant age.

The movement of plants to the data collection system disturbs the growth conditions, may induce mechanical damage, and is limited by the size of the plants. This method works well for small plants and becomes increasingly troublesome, as the plants grow. Similarly, since color information is employed in the image analysis pipeline, stable lighting conditions during image acquisition are required. Because of inconsistent lighting in fields or growth chambers, *in situ* measurements are not possible.

For this study, approximately 120 watermelon samples from four varieties were used. To develop more robust classification and regression models, more varieties are needed.

## Conclusion

This study established that it is possible to classify cold-stressed watermelon plants from normal ones and predict phenotypic traits such as the number of leaves and plant age using selected morphological features from image analysis. The classification model achieved a test accuracy of 100% while using features from two and three different view images, indicating a minimum requirement of two images for 100% classification. An analysis of the few select features used for model development established that texture features and region properties (related to shape and size) were the most important features for classifying normal from stressed watermelon plants.

The models developed for additional phenotypic traits, i.e., plant age and number of leaves, achieved good prediction performance. Overall, this study was able to determine that it is possible to use image analysis to extract morphological features and accurately predict the stress condition and some key phenotypic traits for watermelon plants. This study can serve as a basis for the development of a real-time system for monitoring watermelon plants in high-throughput plant phenotyping facilities. Further studies can be carried out to develop wide-range models for the prediction of multiple phenotypic traits, which would be advantageous for high-throughput phenotyping systems.

## Data Availability Statement

The original contributions presented in the study are included in the article/supplementary material, further inquiries can be directed to the corresponding author.

## Author Contributions

B-KC contributed to the conceptualization, funding acquisition, and supervision of the study and made substantial contributions to the revision of the manuscript. SN, CW, MF, MA, MK, and IB performed the data acquisition and analysis. SN and CW wrote the manuscript. All authors contributed to the article and approved the submitted version.

## Conflict of Interest

The authors declare that the research was conducted in the absence of any commercial or financial relationships that could be construed as a potential conflict of interest.

## Publisher’s Note

All claims expressed in this article are solely those of the authors and do not necessarily represent those of their affiliated organizations, or those of the publisher, the editors and the reviewers. Any product that may be evaluated in this article, or claim that may be made by its manufacturer, is not guaranteed or endorsed by the publisher.
